# Detection of Monkeypox Disease from Human Skin Images with a Hybrid Deep Learning Model

**DOI:** 10.3390/diagnostics13101772

**Published:** 2023-05-17

**Authors:** Fatih Uysal

**Affiliations:** Department of Electrical and Electronics Engineering, Faculty of Engineering and Architecture, Kafkas University, Kars TR 36100, Turkey; fatih.uysal@kafkas.edu.tr; Tel.: +90-534-022-6128

**Keywords:** artificial intelligence, deep learning, image classification, monkeypox disease

## Abstract

Monkeypox, a virus transmitted from animals to humans, is a DNA virus with two distinct genetic lineages in central and eastern Africa. In addition to zootonic transmission through direct contact with the body fluids and blood of infected animals, monkeypox can also be transmitted from person to person through skin lesions and respiratory secretions of an infected person. Various lesions occur on the skin of infected individuals. This study has developed a hybrid artificial intelligence system to detect monkeypox in skin images. An open source image dataset was used for skin images. This dataset has a multi-class structure consisting of chickenpox, measles, monkeypox and normal classes. The data distribution of the classes in the original dataset is unbalanced. Various data augmentation and data preprocessing operations were applied to overcome this imbalance. After these operations, CSPDarkNet, InceptionV4, MnasNet, MobileNetV3, RepVGG, SE-ResNet and Xception, which are state-of-the-art deep learning models, were used for monkeypox detection. In order to improve the classification results obtained in these models, a unique hybrid deep learning model specific to this study was created by using the two highest-performing deep learning models and the long short-term memory (LSTM) model together. In this hybrid artificial intelligence system developed and proposed for monkeypox detection, test accuracy was 87% and Cohen’s kappa score was 0.8222.

## 1. Introduction

Monkeypox is a type of zootonic virus that first emerged through transmission from animals to humans. There appear to be two different lineages of this virus, a west African lineage and a central African lineage. There are animal species that are susceptible to this double-stranded DNA virus. These include tree and rore squirrels, dormice and Gambian pouched rats. Monkeypox is a serious global health problem, affecting the rest of the world in addition to West and East Africa, where its genetic lineage is found. Although it originated in animals, it can also be transmitted from person to person through respiratory secretions and skin lesions during travel. So far, monkeypox has been reported in many countries including Nigeria, Israel, Singapore, Singapore, the United States and the United Kingdom, in addition to Africa, where it first emerged. With monkeypox, it generally takes between 6 and 13 days after infection for symptoms to appear. The infection is divided into two parts: invasion period and skin eruption. In the invasion period, back pain, intense headache, fever, etc., are observed and this lasts between 0 and 5 days. In skin eruption, the appearance of fever varies between 1 and 3 days. Depending on factors such as the health status of the patient and the duration of exposure to the virus, the duration of symptoms in monkeypox, where severe cases are mostly seen in children, is between 2 and 4 weeks. Case fatality rates are observed to be between 3% and 6% [1].

In addition to monkeypox, chickenpox and measles are among the diseases caused by the virus on the skin. This study uses a 4-class open source dataset of skin images and performs monkeypox detection by multi-class classification with a hybrid artificial intelligence system. The main contributions of this study are listed below.

- Since the open source dataset used in this study, which consists of normal, monkeypox, measles, and chickenpox classes, initially had an unbalanced structure, a balanced dataset was created by equalizing the amount of data in each class with data preprocessing and data augmentation operations.
- In the new augmented dataset, the dataset was randomly divided into 80% train, 10% validation and 10% test for training the deep learning models to be used for monkeypox detection.
- In order to analyze the classification results more accurately, augmentation was performed on the train dataset, while no augmentation was performed on the validation and test datasets.
- First, the classification process was performed using state-of-the-art deep learning models, CSPDarkNet, InceptionV4, MnasNet, MobileNetV3, RepVGG, SE-ResNet and Xception.
- Then, in order to improve the classification results and to develop a unique model, a hybrid deep learning model was created by combining the two models with the highest results from these deep learning models and the long short-term memory (LSTM) model.
- In order to further improve monkeypox detection, a unique hybrid artificial intelligence system was developed with a convolutional neural network (CNN)-based model and a LSTM encoder network.

## 2. Related Works

There are various artificial intelligence studies on the detection of monkeypox disease in the literature. Abdelhamid et al. developed a hybrid algorithm to optimize deep neural networks on a monkeypox-related dataset shared openly on the Kaggle platform. By using transfer learning with deep learning models such as AlexNet, VGG, ResNet, and GoogLeNet, they achieved the highest classification accuracy of 98.8% [2]. Almutairi optimized the hyperparameters of the VGG, Xception and MobileNet deep learning models with the metaheuristic Harris Hawks optimizer algorithm using open source, multi-class and two different datasets including monkoypex, and then performed classification with various machine learning classifiers and obtained the highest accuracy values of 98.09% and 97.75% for the two datasets [3]. Dwivedi et al. used the ResNet and EfficientNet-based deep learning models for monkeypox skin lesion detection and found the highest accuracy value was 87% with the EfficientNetB3 model [4]. Gairola and Kumar obtained an accuracy of 95.55%, one of the highest accuracy values in monkeypox detection using the AlexNet, GoogleNet and VGG deep learning models and various machine learning classifiers on an open source monkeypox dataset [5]. Irmak et al. obtained 91.38% as the highest accuracy value in classification processes using pretrained MobileNetV2 and two VGG deep learning models with different number of layers on open source monkeypox skin image dataset [6]. Using an open source dataset for monkeypox image classification, Khafaga et al. obtained 98.83% accuracy in monkeypox detection using deep convolutional neural network optimized with the AL-Biruni Earth radius stochastic fractal search algorithm in addition to the VGG19, ResNet50, GoogleNet, and AlexNet deep learning models [7]. On a two-class dataset consisting of normal and monkeypox classes, Singh and Songare used the deep learning models InceptionV3, GoogLeNet, ResNet50 and VGG16 and found the highest accuracy value of 88.27% in the GoogLeNet model [8]. Sitaula and Shahi first performed classification with 13 different deep learning models on the monkeypox dataset, and then obtained the best accuracy value of 87.13% for multi-class classification with ensemble learning using Xception and DenseNet169, which are the two best-performing models among these models [9]. Sahin et al. obtained the highest classification accuracy of 91.11% in Mo-bileNetV2 model for monkeypox detection for different epoch values using the ResNet18, MobileNetV2, EfficientNet, NasNetMobile, GoogLeNet, and ShuffleNet pretrained deep learning models. They also developed an Android mobile application with Android Studio using Android SDK 12 and the Java programming language [10]. Ahsan et al. first performed data augmentation on a very small amount of normal and monkeypox images, and then obtained a wide range of accuracy values in many different classification processes with the ResNet, VGG, Xception, NasNet, and EfficientNet deep learning models using three different optimizers [11]. Altun et al. obtained the best results with the hybrid MobileNetV3, which was optimized with an f1 score of 0.98 and an accuracy of 96%, in classification processes performed with the ResNet50, DenseNet121, EfficientNetV2, MobileNetV3, Xcception, and VGG19 deep learning models on a two-class dataset containing monkeypox images [12]. Özşahin et al. used the proposed convolutional neural network model, AlexNet, VGG16 and VGG19 in their detection process on two datasets associated with monkeypox and chickenpox and found the best classification accuracy of 99.6% in the proposed deep learning model [13]. Saleh and Rabie used the binary chimp optimization algorithm on the data collected over the internet and obtained a 98.48% classification accuracy in monkeypox operations with an ensemble model consisting of weighted naive bayes, weighted k-nearest neighbors and long short-term memory deep learning model [14]. Almufareh et al. obtained the highest accuracy of 93% by using the model they proposed and the InceptionV3, ResNet, MobileNetV2, EfficientNet deep learning models on two different open source monkeypox skin image datasets [15]. Using the open source monkeypox dataset by Al-rusaini, the highest accuracy value was obtained in the VGG16 model with 96% in the classification processes performed with the support vector machine, ResNet50, SqueezeNet, VGG16 and InceptionV3 models [16]. In the classification process performed by Ariansyah et al. using a dataset containing monkeypox, measles and normal classification, the highest accuracy in the VGG models with the proposed convolutional neural network was achieved in the VGG16 model [17]. VGG16, ResNet50, MobileNetV1, InceptionV3, Xception models were used both alone and as feature extractors in various machine learning classifiers for classification operations on a dataset consisting of normal, monkeypox, measles and chickenpox classes by Bala et al. and also a model called MonkeyNet has been proposed within the scope of this study [18]. Çelik and Özkan performed many classification operations with pretrained VGG, EfficientNet, MobileNet and GoogleNet models on a multi-class dataset, including monkeypox images, and achieved the highest accuracy in the EfficientNet model with the original dataset and in the MobileNet model in the augmented dataset [19]. The highest accuracy value was obtained as 98.8% with Xception, VGG16, VGG19 and modified fine-tuned ResNet50 models for monkeypox detection by Gupta et al. and a secured blockchain-enabled framework was proposed [20]. For monkeypox detection, 93.39% accuracy was achieved by Pramanik et al., by proposing beta normalization-based ensemble learning framework using the InceptionV3, Xception and DenseNet169 deep learning models [21]. Thieme et al. developed a web-based app for the classification of skin lesions caused by monkeypox virus infection using a large number of monkeypox datasets, and 0.91 sensitivity and 0.898 specificity values were obtained in the test dataset with the pretrained ResNet34 deep learning model [22]. On an open-source monkeypox dataset, Velu et al. performed classification with the EfficientNet model and then compared with the reinforcement learning approach Policy Gradient, Actor–Critic, Deep Q-learning network and Double Deep Q-learning network, the highest accuracy was achieved as 0.985 [23]. For the detection of monkeypox disease by Yasmin et al., using DenseNet201, EfficientNetB7, Inception-ResNetV2, InceptionV3, VGG16, and ResNet50 models, the highest accuracy was obtained in the InceptionV3 model, and a fine-tuned version of this model was recommended, and 100% accuracy in the new model called PoxNet22 was achieved [24].

It is observed that studies in the literature often use deep learning models such as AlexNet, VGG, and ResNet for monkeypox detection on multi-class, mostly two-class, datasets and also use machine learning models for classification. This study develops a novel hybrid artificial intelligence system for monkeypox detection on an open source, four-class dataset using state-of-the-art deep learning models and the LSTM model, which has not been used so far in the literature.

In Section 3, the details of the monkeypox dataset used in this study, the data augmentation and data preprocessing applied to this dataset, and the deep learning models used for classification are described. Section 4 describes the proposed hybrid model, evaluation metrics and detailed classification results. In Section 5, the results obtained for monkeypox detection are analyzed and interpreted, the main contributions of this study and its differences from the literature are emphasized, and what improvements could be made in the future following the current study are stated.

## 3. Materials and Methods

**Figure 1:**
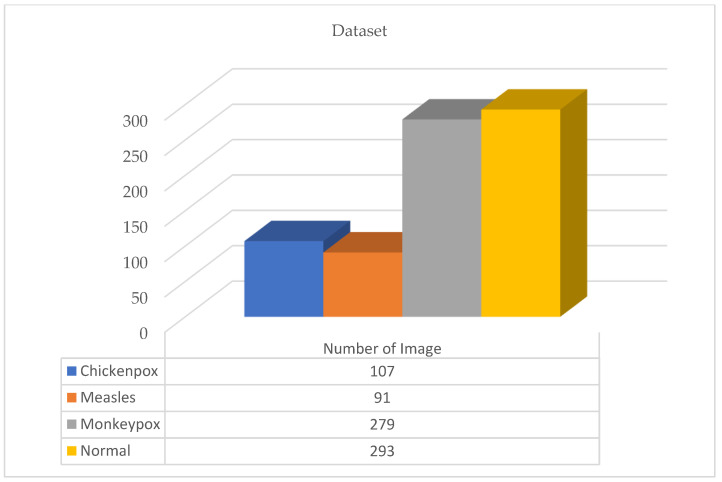
Original first version of the dataset.

**Figure 2:**
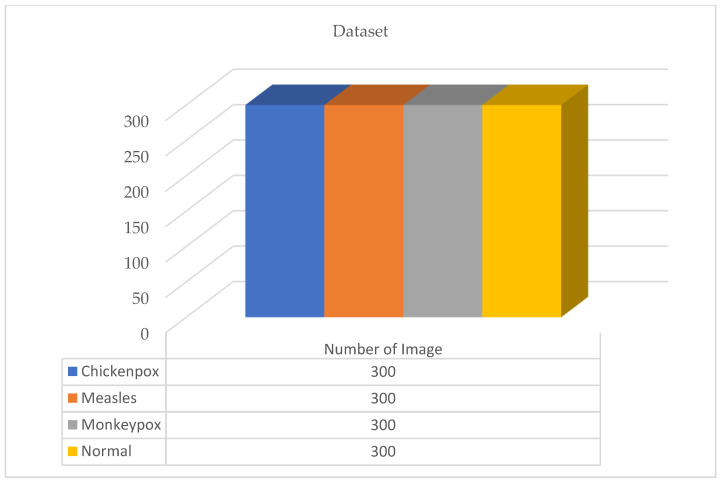
The new version of the dataset after data augmentation.

The dataset used in this study for monkeypox detection is an open source shared dataset through the Kaggle platform [25]. The dataset consists of normal, monkeypox, measles and chickenpox classes. It is understood that the distribution in the dataset is unbalanced. However, in artificial intelligence models used in classification problems, the class distribution should be as balanced as possible in order to fully realize network training. For this reason, various data augmentation operations were first performed on the dataset. These augmentations are equalize, horizontal flip, random brightness contrast, hue saturation value, shift scale rotate and RGB shift. The parameters and values of the data augmentations are given in Table 1. Additionally, Figure 1 and Figure 2 show the first version of the dataset and the new version after augmentation, respectively.

**Figure 3:**
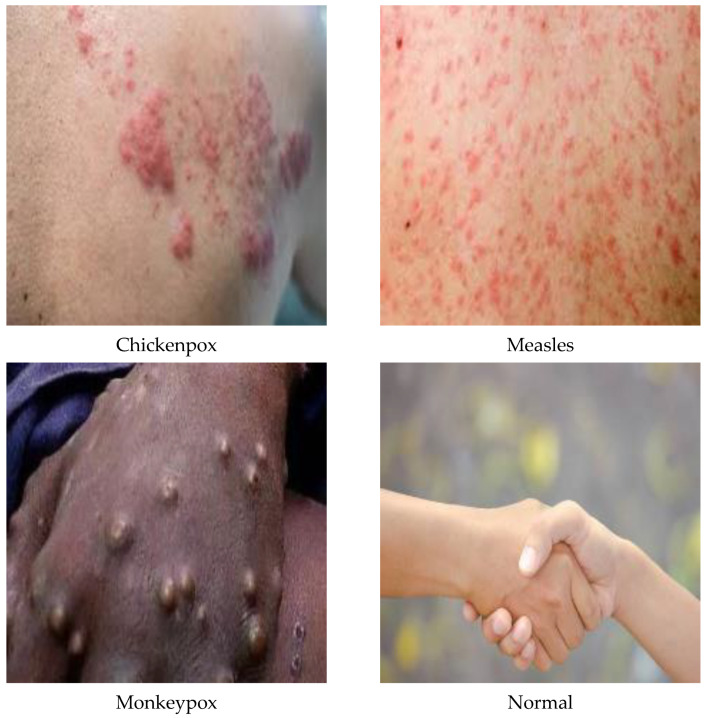
A sample of the classes of the dataset in its original state.

**Figure 4:**
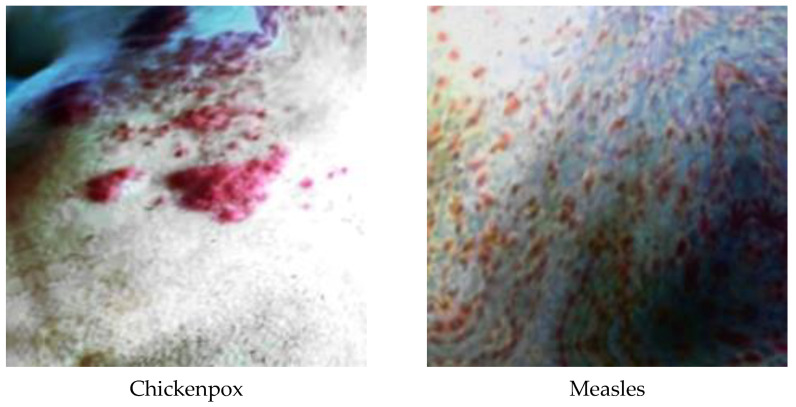

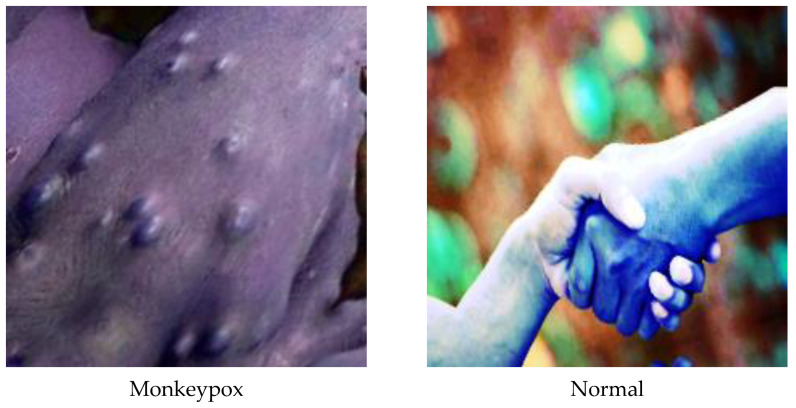
A sample of classes after dataset preprocessing.

A total of 770 skin image datasets are available in the initial version of the dataset, including 293 normal, 279 monkeypox, 91 measles and 197 chickenpox images. Before data augmentation, a total of 240 images, 60 from each class in the original dataset, were selected for use in the test and validation dataset. Data augmentation was applied to the remaining images from the original dataset and a train dataset containing 960 images was obtained. Thanks to this method, the images in the test and validation set are not included in the train dataset. In this way, the success of this study and the designed models were handled in a more realistic way. After the data augmentation operations obtained by performing data preprocessing, a new dataset with a total of 1200 skin images, 300 in each class, was obtained. A sample image of both the original images and the images after data processing for each class of the dataset used in this study are given in Figure 3 and Figure 4, respectively.

**Figure 5:**
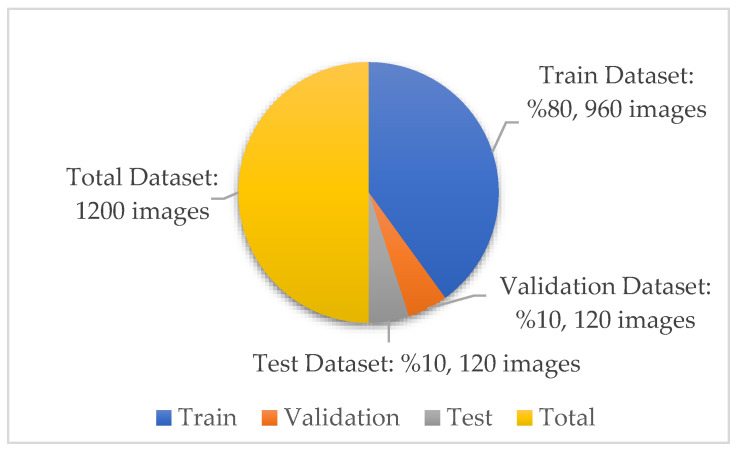
Dataset quantity and distribution.

In the new version of the dataset with data augmentation and data preprocessing, the training, validation and test distributions required for network training and classification in deep learning models are 80%, 10% and 10%, respectively. The images in each class were randomly determined in this data percentage distribution. No splitting occurred in the augmented dataset. A total of 30 test and 30 validation images were randomly selected for each class from the original dataset. The purpose of the random selection is that the researcher does not have the images in the test and validation dataset relatively easily. After this step, a test and validation dataset containing 120 images in total was obtained. A training dataset containing 960 images is required to ensure 80% training, 10% validation and 10% testing. Therefore, these 960 training datasets were obtained by augmenting the remaining 530 images in the original dataset. There is no imbalance as the test dataset contains 30 images from each class. Information on the amount and distribution of the data for each class is also shown in Figure 5 below.

In the open source dataset used in this study, there are 300 images for each class—240 in the training data, 30 in the test data, and 30 in the validation data. A total of 960 images were used for training in the dataset. No augmentation was made to analyze the classification results performed with the test dataset more realistically and accurately. Since the dataset distribution was determined as 80%, 10%, and 10%, the size of the training dataset was determined in this way.

First of all, a total of 7 different state-of-the-art deep learning models were used: CSPDarkNet with 53 layers, MnasNet with 100 layers, SE-ResNet with 50 layers, Xcep-tion with 71 layers, and InceptionV4, MobileNetV3, and RepVGG with different layer values. In addition to using these deep learning models for classification, a unique hybrid model was created by combining the best two CNN models with the LSTM model. All deep learning models that were customized and used in the classification process are given below as subheadings.

### 3.1. CSPDarkNet

DarkNet is a convolutional neural network used as a backbone in the YOLO object detection model. This backbone, which contains 3 × 3 and 1 × 1 convolutional layers, has different types depending on the number of layers [26]. Cross Stage Partial Network (CSPNet) is a backbone that can be applied in many different deep learning models and makes the model lightweighted [27]. In the YOLOv4 object detection model, CSPDarkNet with 53 layers was used as the backbone [28]. In addition to being used as a backbone in object detection models, it is also used in classification problems since it is a convolution neural network. In this study, CSPDarkNet-53 model is used for monkeypox detection by modifying the last layer.

### 3.2. InceptionV4

InceptionV4 is a convolutional neural network with more inception modules compared to its predecessor InceptionV3. InceptionV4 is an inception variant of the hybrid inception version Inception-ResNetV2 which does not include residual connections [29]. InceptionV4 model architecture used in this study was used for monkeypox detection.

### 3.3. MnasNet

MnasNet is a convolutional neural network whose main building block is the in-verted residual block in MobileNetV2 and proposes an automated mobile neural architecture search approach [30]. The MnasNet model used in this study has 100 layers and the number of features in the last layer is adapted for multi-class classification in accordance with the monkeypox dataset classes.

### 3.4. MobileNetV3

MobileNetV3 is a convolutional neural network that boasts an efficient design incorporating squeeze-and-excitation modules, making it suitable for various tasks such as classification, segmentation, and detection. This network has two variants, MobileNetV3-Large and MobileNetV3-Small, which cater to different levels of resource usage. On the ImageNet dataset for classification and the COCO dataset for detection, MobileNetV3 demonstrates improved performance compared to its predecessor, MobileNetV2 [31]. In this study, the MobileNetV3-Large model with 100 layers was adapted and used for monkeypox detection.

### 3.5. RepVGG

RepVGG is fundamentally a deep learning model that employs 3 × 3 convolution layers and ReLU non-linear activation functions. It features two primary types, RepVGG-A and RepVGG-B, each with distinct subtypes corresponding to the layers within each stage [32]. The RepVGG-B0 model, with its varying number of layers among the subtypes, was adapted to accommodate the specific task of monkeypox detection in this current study.

### 3.6. SE ResNet

SE ResNet is a variant of the ResNet model and is a deep learning model that in-cludes squeeze-and-excitation blocks. This model, which uses the SE ResNet module instead of the original ResNet module, gives better classification performance than many models on the ImageNet dataset [33]. A modified SE ResNet model architecture was used for monkeypox detection. In monkeypox detection using the 50-layer SE ResNet model, the number of features was reduced to 4 in the last layer in accordance with the multi-class classification and the number of classes was equalized.

### 3.7. Xception

The Xception model is a convolutional neural network that includes depthwise separable convolution layers instead of the inception module and uses model parameters more efficiently compared to the InceptionV3 model. The Xception deep learning model, which stands out with its better performance than the InceptionV3 model, especially on the ImageNet database, can be used for many image classification problems [34]. In this study, Xception is used by modifying the last layer to generate an output with 4 classes suitable for monkeypox detection.

### 3.8. LSTM

The LSTM model is a deep learning model, which is a type of recurrent neural networks. Its basic architecture consists of input, recurrent LSTM and output layers, respectively. LSTMs actually address the vanishing gradient problem. The recurrent connections in the LSTM layer are cyclic [35,36]. In this study, the LSTM model is used as an encoder network immediately after the CNN structure in the developed hybrid model. The architectural details of the LSTM used are described in detail in the experiments section.

## 4. Experiments

**Figure 6:**
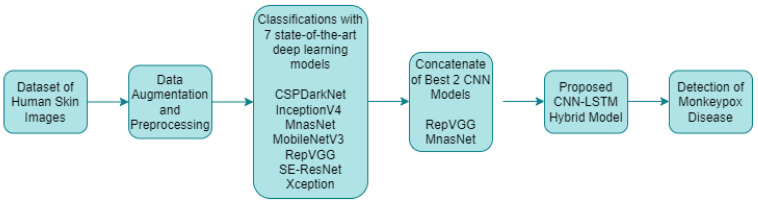
Proposed approach for monkeypox disease detection.

In the classification studies for monkeypox detection, seven different deep learning models with different layers and structures were used alone. The training process was carried out in this study by adapting pretrained deep learning models that utilized transfer learning from the ImageNet dataset. The initial 1000-class structure in the final layers was transformed to a four-class configuration, tailored to the dataset employed in the current research. After data augmentation and preprocessing, the results of these classification processes were analyzed and the best two CNN models were determined. These models were combined with a LSTM encoder network and a hybrid artificial intelligence system for monkeypox detection was developed. The proposed approach for monkeypox detection is presented in Figure 6 below.

**Figure 7:**
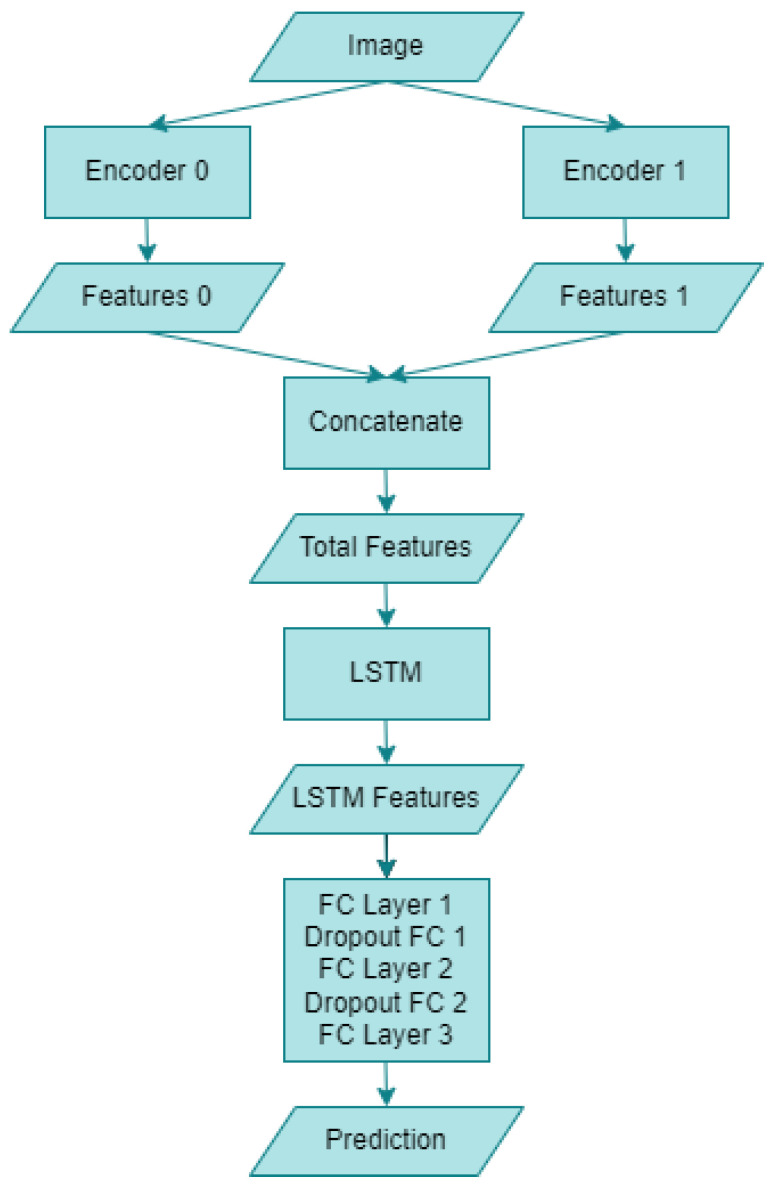
Block diagram of the proposed hybrid artificial intelligence system.

The block diagram of the hybrid artificial intelligence system proposed within the scope of this study is given in Figure 7 below. The “Image” section refers to human skin images utilized in this research. Following the necessary augmentation and preprocessing of the dataset images, they are fed into two distinct encoders. “Encoder 0” corresponds to the RepVGG-B0 deep learning model, whereas “Encoder 1” denotes the MnasNet-100 deep learning model. Upon entering the artificial intelligence system, the two encoders yield “Features 0” and “Features 1”, comprising 1280 features for RepVGG and MnasNet, respectively. Subsequently, a concatenation operation is performed on both models’ features, resulting in 2560 combined features, as indicated in the “Total Features” section. This novel CNN encoder structure is then integrated with an LSTM model. Following the LSTM outputs, referred to as “LSTM Features”, a “Dropout FCs” layer with a ratio of 0.1 is connected to the “FC Layer”. Finally, the monkeypox detection process is executed through the “Prediction” output. The structure of the proposed hybrid model is further detailed in Algorithm 1 below.
**Algorithm 1** Proposed CNN–LSTM Hybrid Model**Input:** test_dataset **Process:**  for image in test_dataset:   features_0 = Model_I (image)   features_1 = Model_II (image)   total_features = concat (features_0, features_1)   features_lstm = LSTM (total_features)   out = nn.Linear (1024, 256) (features_lstm)   Dropout (0.1)   out = nn.Linear (256, 128) (features_lstm)   Dropout (0.1) prediction = nn.Linear (128, num_classes) (features_lstm) **Output:** prediction

The operation of the above proposed algorithm is as follows: Images from any dataset are sent to both CNN architectures, respectively, and two different feature maps are obtained. Then, a single vector is obtained by combining both feature maps. This feature vector obtained is given as an input to an LSTM network and the LSTM network is provided to perform a feature extraction. The final feature vector obtained is passed through two layers and the classification process is performed.

Classification was performed using the Google Colab environment. All classifications in Colab are based on PyTorch, an open source machine learning framework. In addition, torch was used for the LSTM model, timm [37] for CNN encoder, albumentations [38] for data augmentation, and splitfolders for dataset generation. The parameters used in all artificial intelligence models for monkeypox detection are learning rate 0.001, epoch 100, batch size 8, optimizer Adam, loss function cross entropy loss.

### 4.1. Evaluation Metrics

There are many evaluation metrics in the literature to clearly evaluate the results obtained in binary and/or multi-class classification problems. In order to accurately analyze the results of multi-class classification for monkeypox detection, many possible evaluation metrics have been obtained in this study. These metrics are confusion matrices consisting of true-positive (TP), false-positive (FP), true-negative (TN) and false-negative (TN) values for each class; precision, recall, f1 score, ROC curve, AUC score obtained for each class; and accuracy, Cohen’s kappa score and Matthews correlation coefficient score obtained using training, validation and test data. Equations (1)–(9) were taken into account in the calculation of all metrics.

(1) $$\mathrm{Accuracy}={\;p}_{0}=\frac{\mathrm{TP}+\mathrm{TN}}{\mathrm{TP}\;+\;\mathrm{TN}\;+\;\mathrm{FP}\;+\;\mathrm{FN}}$$

(2) $$p_{\mathrm{positive}}=\frac{\left(\mathrm{TP}\;+\;\mathrm{FP}\right)\left(\mathrm{TP}\;+\;\mathrm{FN}\right)}{{\left(\mathrm{TP}\;+\;\mathrm{TN}\;+\;\mathrm{FP}\;+\;\mathrm{FN}\right)}^{2}}$$

(3) $$p_{\mathrm{negative}}=\frac{\left(\mathrm{FN}\;+\;\mathrm{TN}\right)\left(\mathrm{FP}\;+\;\mathrm{TN}\right)}{{\left(\mathrm{TP}\;+\;\mathrm{TN}\;+\;\mathrm{FP}\;+\;\mathrm{FN}\right)}^{2}}$$

(4) $$p_{e}=p_{\mathrm{positive}}+p_{\mathrm{negative}}$$

(5) $${\mathrm{Cohen}}^{’}s\;\mathrm{kappa}=\frac{p_{0}-p_{e}}{1-p_{e}}$$

(6) $$\mathrm{Matthews}\;\mathrm{correlation}=\frac{\left(\mathrm{TN}*\mathrm{TP}\right)-\left(\mathrm{FP}*\mathrm{FN}\right)}{\sqrt{\left(\mathrm{TN}+\mathrm{FN}\right)\left(\mathrm{FP}+\mathrm{TP}\right)\left(\mathrm{TN}+\mathrm{FP}\right)\left(\mathrm{FN}+\mathrm{TP}\right)}}$$

(7) $$\mathrm{Precision}=\frac{\mathrm{TP}}{\mathrm{TP}+\mathrm{FP}}$$

(8) $$\mathrm{Recall}=\frac{\mathrm{TP}}{\mathrm{TP}+\mathrm{FN}}$$

(9) $$F1\;\mathrm{score}=\frac{2\mathrm{TP}}{2\mathrm{TP}+\mathrm{FP}+\mathrm{FN}}$$

### 4.2. Monkeypox Detection Results of Deep Learning Models

**Figure A1:**
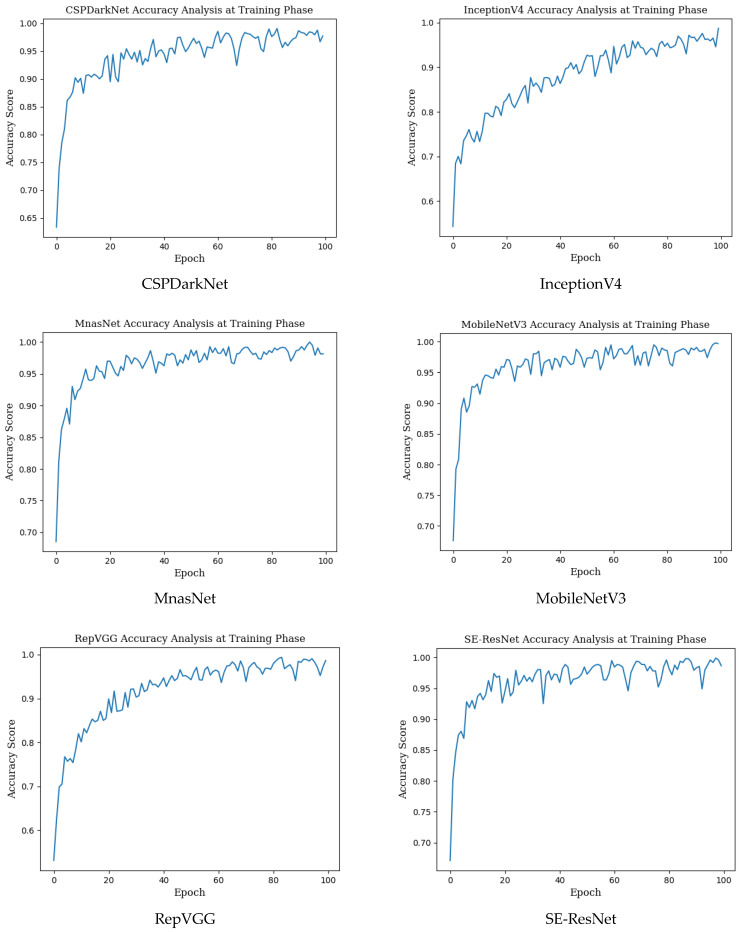

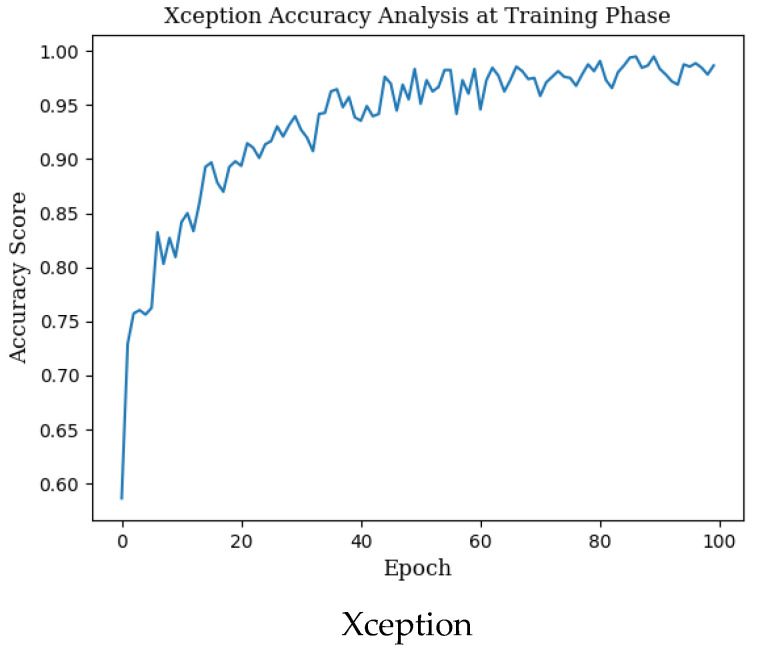
Epoch change graphs of accuracy for training.

The mean accuracy with standard deviation (±SD), highest accuracy, Cohen’s kappa, and Matthews correlation coefficient (MCC) scores obtained in the training phase for seven different state-of-the-art deep learning models used in monkeypox detection and the precision, recall, f1 score and AUC score values in the monkeypox class are given in Table 2 below. Epoch change graphs of accuracy for training are included in Figure A1 in Appendix A.

**Figure A2:**
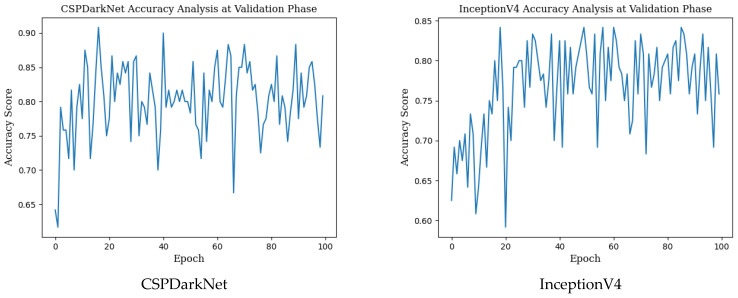

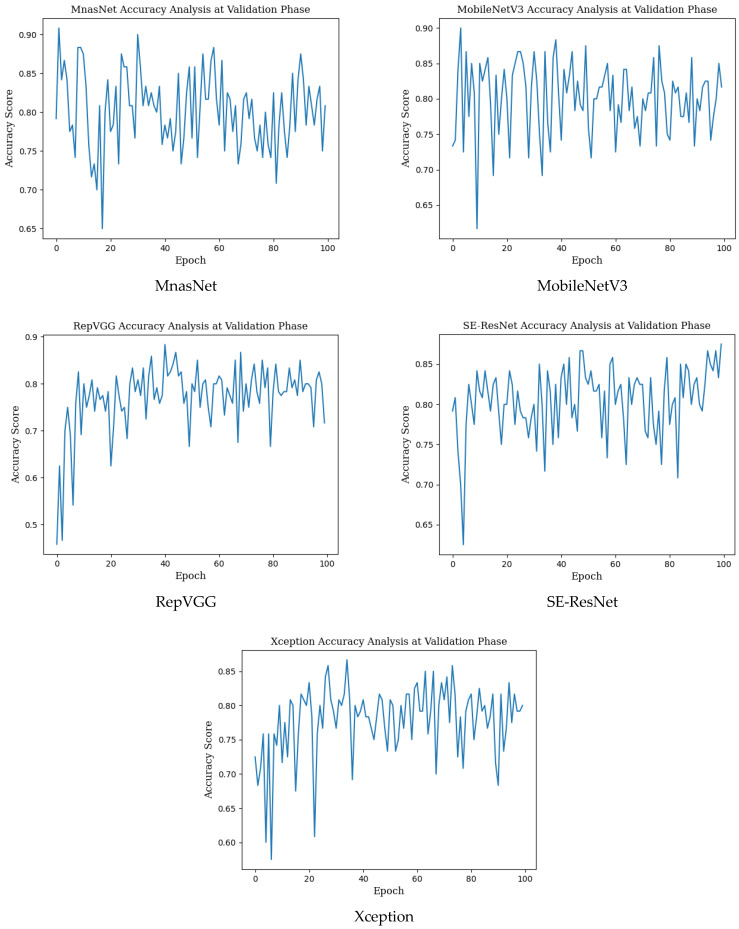
Epoch change graphs of accuracy for validation.

The training results in the table above show that network training was performed in the best way in the MnasNet model with the highest accuracy value. The precision, recall, f1 score and AUC score values and mean accuracy with standard deviation (±SD), highest accuracy, Cohen’s kappa, Matthews correlation coefficient scores in the monkeypox class obtained for the validation phase in deep learning models used for monkeypox detection are given in Table 3 below. Epoch change graphs of accuracy for validation are included in Figure A2 in Appendix B.

Table 3 shows that the best-performing models are CSPDarkNet and MnasNet for accuracy, Cohen’s kappa and Matthews correlation coefficient scores. Best epoch of accuracy for training and validation is included in Table A1 in Appendix C. Table 4 shows the accuracy with standard deviation (±SD), Cohen’s kappa, Matthews correlation coefficient scores for the classifications performed on the test data after training and validation, as well as the precision, recall, f1 score and AUC score values in the monkeypox class.

**Figure 8:**
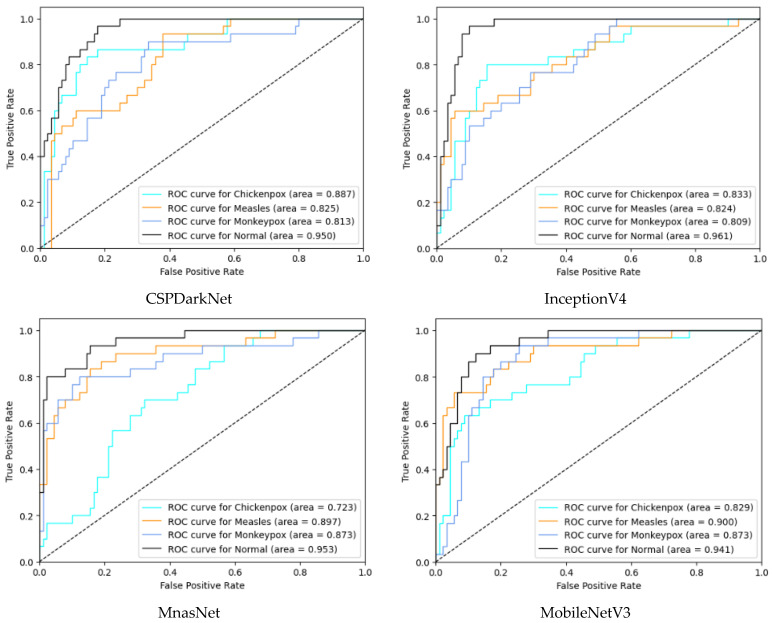

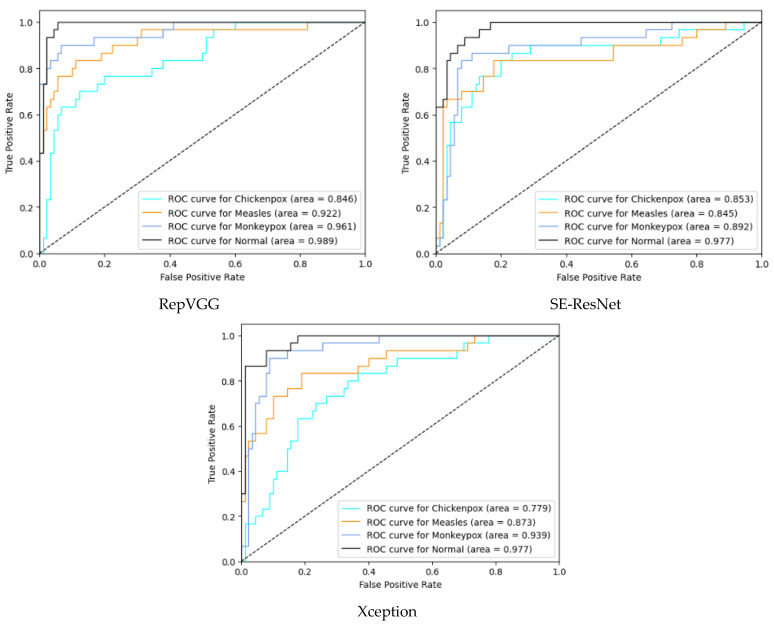
ROC curves in test (ROC: Receiver Operator Characteristic).

In the multi-class classification process for monkeypox detection, the highest accuracy values among seven different deep learning models were obtained as 0.85 in RepVGG and 0.84 in MnasNet. The ROC curves obtained for each class with deep learning models on the test dataset are given in Figure 8 below.

**Figure 9:**
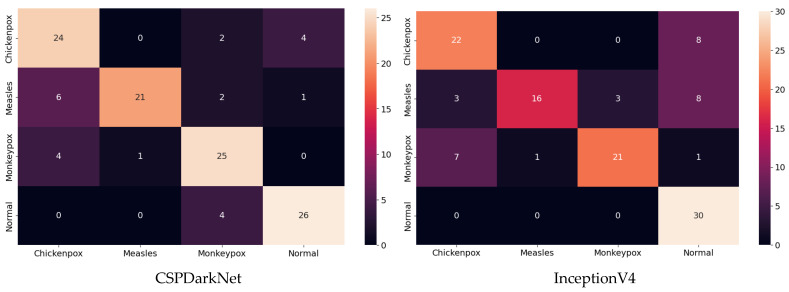

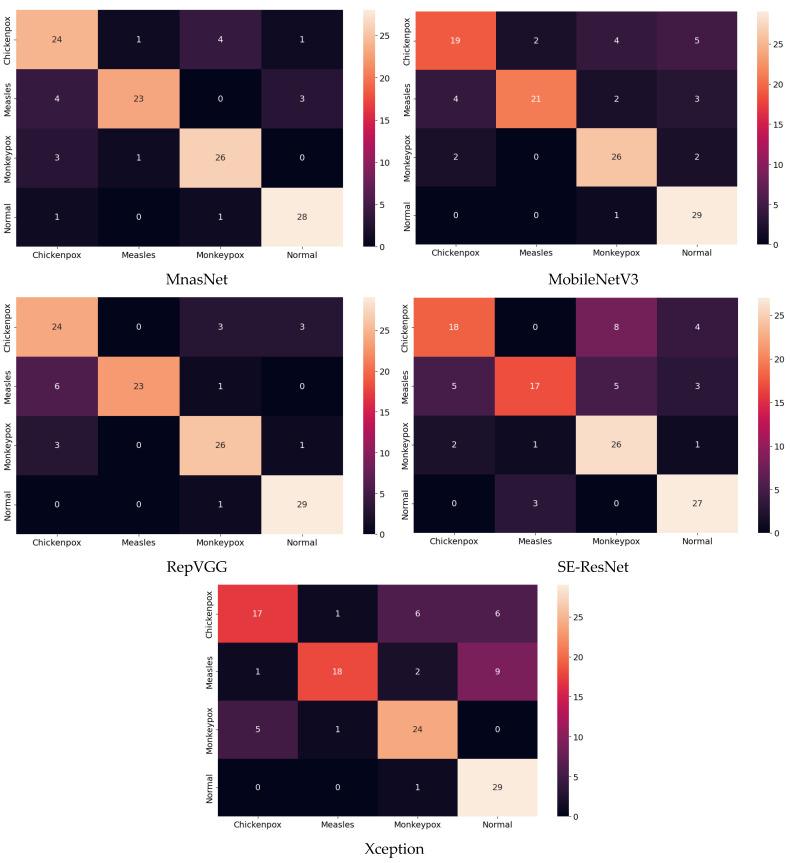
Confusion matrices in test.

Among the deep learning models used in classification, the ROC curves in the monkeypox class show that the two highest AUC scores are in the RepVGG and Xception models. The confusion matrices obtained for the test dataset are given in Figure 9 below.

The classification results obtained using the test dataset show that the models to be used in the CNN part of the hybrid model should be RepVGG and MnasNet to further improve classification accuracy.

### 4.3. Monkeypox Detection Results of the Proposed Hibrid Deep Learning Model

**Figure 10:**
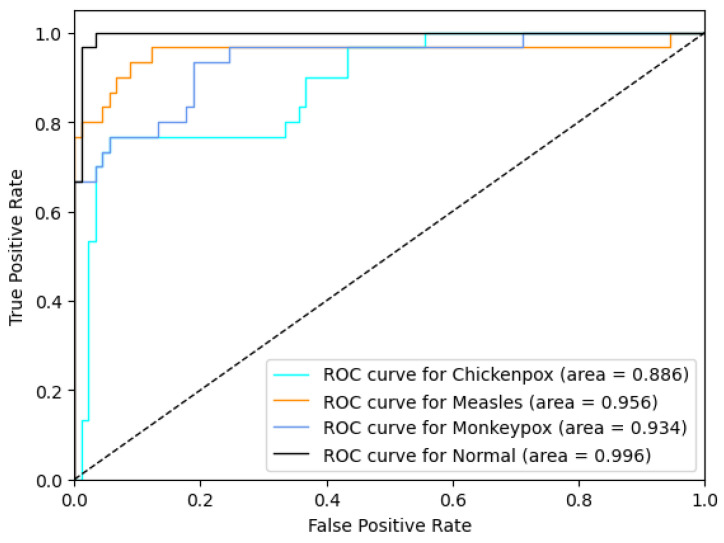
ROC curve in the proposed hybrid model (ROC: Receiver Operator Characteristic).

**Figure 11:**
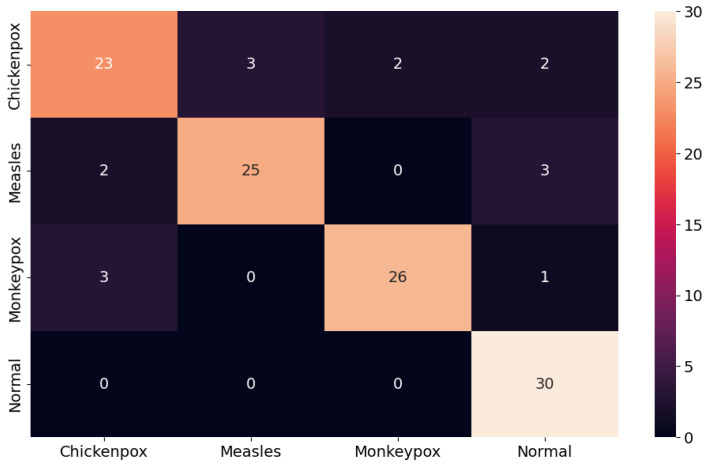
Confusion matrix in the proposed hybrid model.

The proposed CNN–LSTM hybrid deep learning model for monkeypox detection achieved the following scores on the test dataset: 0.87 accuracy, 0.8222 Cohen’s kappa, and 0.8240 Matthews correlation coefficient score. Furthermore, for the monkeypox class, the model attained 0.93 precision, 0.87 recall, 0.90 f1 score, and 0.9344 AUC score values. Below, Figure 10 shows the ROC curve for the proposed hybrid deep learning model and Figure 11 shows the confusion matrix.

**Figure 12:**
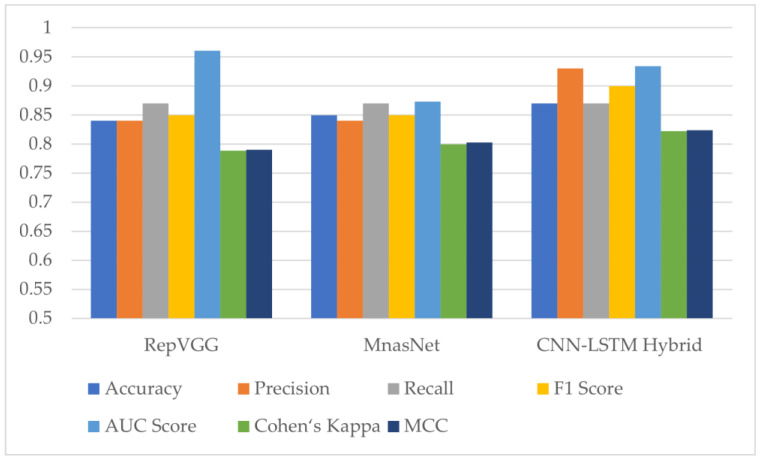
Test results of RepVGG, MnasNet and the proposed hybrid deep learning model (AUC: Area Under the ROC (Receiver Operator Characteristic) Curve, MCC: Matthews Correlation Coefficient).

Two deep learning models, RepVGG and MnasNet, which produced the highest results among the seven different models employed for monkeypox detection, were utilized in the proposed hybrid deep learning model within the scope of this study. The evaluation metric results for the test dataset can be found in Figure 12 and Table 5 below. The results show an increase in accuracy, Cohen’s kappa and Matthews correlation coefficient scores with the hybrid model.

There are many independent variables such as the dataset used in studies on similar subjects, batch sizes and image sizes that change depending on the performance of the devices used during model training, and hyper parameters (optimizer, learning rate, mini batch size) preferred during model training. In two different studies using the same model, different results can be achieved by using different batch sizes. However, this does not mean that one of the models is worse. In this context, since the classification results obtained depend on the dataset, it is more appropriate to evaluate it in itself. In this study, it was found that hybrid models achieve higher performance than conventional models.

## 5. Conclusions and Future Works

In this study, firstly, data augmentation and preprocessing operations were per-formed on open source and 4-class human skin images in order to make the dataset balanced. In the created balanced dataset, classification was performed with seven different pretrained deep learning models. Each of these various deep learning models used in this study was used pretrained in ImageNet. The structure, which has 1000 classes in ImageNet, has been made into 4 classes to be suitable for operation. While machine learning algorithms and traditional neural networks process the image as a single input, convolutional neural networks use a moving filter to allow the model to learn local features such as edges and corners. Convolutional neural network architectures can have a very deep structure, containing tens or even hundreds of layers, making it easier to learn complex features in the data compared to other methods. Therefore, better results were obtained using convolutional neural networks in this study. The results obtained were analyzed and a hybrid deep learning model was created by using the best two CNN models and LSTM encoder together in order to further improve monkeypox detection. A 140-layer RepVGG-B0 and a 100-layer MnasNet100 were used in the CNN part of the CNN–LSTM hybrid model proposed in this study. In the LSTM part, there are four layers. The final classifier network of the hybrid model consists of two layers. With this hybrid artificial intelligence system created for monkeypox disease detection, the highest classification results were obtained, 0.87, 0.8222 and 0.8240 in test accuracy, Cohen’s kappa and Matthews correlation coefficient scores, respectively. Since hybrid systems are designed by combining different types of models, they can learn more generalizable features and thus overfitting is prevented. Likewise, combining different architectures gives reliable results with higher accuracy for the problem being dealt with. For this reason, a hybrid artificial intelligence system was used in this study. The contributions of this study to the literature are listed below.

- In order to analyze the classification results correctly, the imbalance in the dataset was eliminated with various data augmentation methods and the dataset was balanced.
- The augmentation procedures for the new balanced dataset were applied only to the training dataset. Thus, since the validation and test datasets were in its original state, the evaluation metrics obtained in the classification could be analyzed in a more realistic way.
- In order to detect monkeypox, many different state-of-the-art deep learning models were used, adapted to multi-class classification.
- The classification results of deep learning models with different layers and structures were analyzed with many different evaluation metrics and the two most appropriate CNN models were determined.
- A study-specific hybrid deep learning model was developed with CNN models and LSTM encoder models.
- With the proposed CNN–LSTM hybrid artificial intelligence system, the highest test accuracy, Cohen’s kappa and Matthews correlation coefficient scores in monkeypox detection were obtained.

In future studies, machine learning models can be utilized for monkeypox disease detection alongside the deep learning models used in this study and the hybrid model developed in this study. In addition to the multi-class classification, which is a more comprehensive classification problem, binary classifications can be performed for different human skin diseases. In the future, an online web interface, an offline graphical user interface and/or a mobile application for monkeypox detection can be developed for real-time use by physicians.

## Figures and Tables

**Table 1:** Data augmentation types and parameters (*p* = probability).

Types	Parameters	Types	Parameters
Equalize	*p* = 0.5	Shift Scale Rotate	shift_limit = 0.1
Horizontal Flip	*p* = 0.7		scale_limit = 0.05
Random Brightness Contrast	brightness_limit = 0.1		rotate_limit = 60
	contrast_limit = 0.5		*p* = 0.7
	*p* = 0.5	RGB Shift	r_shift_limit = 5
Hue Saturation Value	hue_shift_limit = 20		g_shift_limit = 5
	sat_shift_limit = 30		b_shift_limit = 5
	val_shift_limit = 20		*p* = 0.2
	*p* = 0.5		

**Table 2:** Training results of deep learning models (SD: Standard Deviation, AUC: Area Under the ROC (Receiver Operator Characteristic) Curve, MCC: Matthews Correlation Coefficient).

Model Name	Mean Accuracy (±SD)	Highest Accuracy	Precision	Recall	F1 Score	AUC Score	Cohen’s Kappa	MCC
CSPDarkNet	0.9417 (±0.0525)	0.9906	0.96	0.90	0.92	0.911	0.9263	0.9268
InceptionV4	0.8808 (±0.0821)	0.9875	0.98	0.80	0.88	0.865	0.8888	0.8905
MnasNet	0.9645 (±0.0410)	1.0000	0.94	0.84	0.89	0.917	0.875	0.8761
MobileNetV3	0.9615 (±0.0435)	0.9979	0.99	0.89	0.94	0.877	0.95	0.9506
RepVGG	0.9176 (±0.0828)	0.9938	0.96	0.88	0.92	0.984	0.9166	0.9181
SE-ResNet	0.9624 (±0.0439)	0.9990	0.99	1.00	0.99	0.997	0.9847	0.9847
Xception	0.9330 (±0.0705)	0.9948	1.00	0.95	0.98	0.984	0.9805	0.9806

**Table 3:** Validation results of deep learning models (SD: Standard Deviation, AUC: Area Under the ROC (Receiver Operator Characteristic) Curve, MCC: Matthews Correlation Coefficient).

Model Name	Mean Accuracy (±SD)	Highest Accuracy	Precision	Recall	F1 Score	AUC Score	Cohen’s Kappa	MCC
CSPDarkNet	0.8023 (±0.0534)	0.9083	0.89	0.83	0.86	0.843	0.8777	0.8789
InceptionV4	0.7670 (±0.0572)	0.8417	0.82	0.93	0.87	0.860	0.7888	0.7957
MnasNet	0.8027 (±0.0483)	0.9083	0.90	0.90	0.90	0.929	0.8777	0.8778
MobileNetV3	0.8010 (±0.0502)	0.9000	0.88	0.97	0.92	0.930	0.8666	0.8701
RepVGG	0.7714 (±0.0711)	0.8833	0.88	0.93	0.90	0.980	0.8444	0.8470
SE-ResNet	0.8043 (±0.0422)	0.8750	0.79	0.90	0.84	0.940	0.8333	0.8373
Xception	0.7782 (±0.0520)	0.8667	0.86	1.00	0.92	0.979	0.8222	0.8270

**Table 4:** Test results of deep learning models (SD: Standard Deviation, AUC: Area Under the ROC (Receiver Operator Characteristic) Curve, MCC: Matthews Correlation Coefficient).

Model Name	Accuracy (±SD)	Precision	Recall	F1 Score	AUC Score	Cohen’s Kappa	MCC
CSPDarkNet	0.80 (±0.0408)	0.76	0.83	0.79	0.813	0.7333	0.7364
InceptionV4	0.74 (±0.0528)	0.88	0.70	0.78	0.809	0.6555	0.6712
MnasNet	0.84 (±0.0348)	0.84	0.87	0.85	0.873	0.7888	0.7901
MobileNetV3	0.79 (±0.0499)	0.79	0.87	0.83	0.873	0.7222	0.7277
RepVGG	0.85 (±0.0290)	0.84	0.87	0.85	0.961	0.8	0.8025
SE-ResNet	0.73 (±0.0295)	0.67	0.87	0.75	0.892	0.6444	0.6508
Xception	0.73 (±0.0396)	0.73	0.80	0.76	0.939	0.6444	0.6552

**Table 5:** Test results of RepVGG, MnasNet and the proposed hybrid deep learning model (SD: Standard Deviation, AUC: Area Under the ROC (Receiver Operator Characteristic) Curve, MCC: Matthews Correlation Coefficient).

Model Name	Accuracy (±SD)	Precision	Recall	F1 Score	AUC Score	Cohen’s Kappa	MCC
RepVGG	0.84 (±0.0290)	0.84	0.87	0.85	0.961	0.7888	0.7901
MnasNet	0.85 (±0.0348)	0.84	0.87	0.85	0.873	0.8	0.8025
Proposed CNN–LSTM hybrid model	0.87 (±0.0352)	0.93	0.87	0.90	0.934	0.8222	0.8240

## Data Availability

Data used in this study are available at: https://www.kaggle.com/datasets/dipuiucse/monkeypoxskinimagedataset (accessed on 1 September 2022).

## Appendix A. Epoch Change Graphs of Accuracy for Training

Epoch change graphs of accuracy for training are given in Figure A1 below. The training accuracies given in Table 2 are the highest values in the training accuracy and epoch change graphs given in Figure A1.

## Appendix B. Epoch Change Graphs of Accuracy for Validation

Epoch change graphs of accuracy for validation are given in Figure A2 below. The validation accuracies given in Table 3 are the highest values in the validation accuracy and epoch change graphs given in Figure A2.

## Appendix C. Best Epoch of Accuracy for Training and Validation

Best epoch of accuracy for training and validation is given in Table A1 below. The training accuracy given in Table 2 and the validation accuracy given in Table 3 are the values corresponding to the best epochs given in Table A1. When the epoch values with the highest accuracy given in this table are examined, it is observed that network training in the training part is completed with a rate close to 100%. When the validation part is examined, it is seen that the highest scores are mostly obtained within the first 50 epochs. Classification processes in the test dataset were carried out using weights at the highest epoch values obtained in validation, and no network training was performed in the test dataset. The independence of the test dataset from the validation dataset made the results more realistic and reliable.

**Table A1:** Best epoch of accuracy for training and validation.

Model Name	Best Epoch of Accuracy for Training	Best Epoch of Accuracy for Validation
CSPDarkNet	83	17
InceptionV4	100	19
MnasNet	95	3
MobileNetV3	99	4
RepVGG	84	41
SE-ResNet	98	100
Xception	87	35
